# ORIC-101, a Glucocorticoid Receptor Antagonist, in Combination with Nab-Paclitaxel in Patients with Advanced Solid Tumors

**DOI:** 10.1158/2767-9764.CRC-24-0115

**Published:** 2024-09-13

**Authors:** Christopher T. Chen, Vishesh Khanna, Shivaani Kummar, Raghad M. Abdul-Karim, David Sommerhalder, Anthony W. Tolcher, Naoto T. Ueno, Sarah Lindsey Davis, Douglas W. Orr, Erika Hamilton, Manish R. Patel, Alexander I. Spira, Shekeab Jauhari, Vaia Florou, Maureen Duff, Rongda Xu, Jian Wang, Shravani R. Barkund, Haiying Zhou, Aleksandr Pankov, Wayne Kong, Nadine S. Jahchan, Erica L. Jackson, Jessica D. Sun, Melissa R. Junttila, Pratik S. Multani, Anneleen Daemen, Edna Chow Maneval, Pamela N. Munster

**Affiliations:** 1 Division of Oncology, Department of Medicine, Stanford University School of Medicine, Palo Alto, California.; 2 Knight Cancer Institute, Oregon Health and Science University, Portland, Oregon.; 3 NEXT Oncology, San Antonio, Texas.; 4 University of Texas MD Anderson Cancer Center, Houston, Texas.; 5 University of Colorado Cancer Center, Aurora, Colorado.; 6 Mary Crowley Cancer Research, Dallas, Texas.; 7 Sarah Cannon Research Institute, Nashville, Tennessee.; 8 Florida Cancer Specialists/Sarah Cannon Research Institute, Sarasota, Florida.; 9 Virginia Cancer Specialists, Fairfax, Virginia.; 10 Florida Cancer Specialists/Sarah Cannon Research Institute, Lake Mary, Florida.; 1 1Huntsman Cancer Institute, University of Utah, Salt Lake City, Utah.; 12 ORIC Pharmaceuticals, South San Francisco, California.; 13 University of California San Francisco Helen Diller Family Comprehensive Cancer Center, San Francisco, California.

## Abstract

**Purpose:**

In preclinical models, glucocorticoid receptor (GR) signaling drives resistance to taxane chemotherapy in multiple solid tumors via upregulation of antiapoptotic pathways. ORIC-101 is a potent and selective GR antagonist that was investigated in combination with taxane chemotherapy as an anticancer regimen preclinically and in a phase 1 clinical trial.

**Patients and Methods:**

The ability of ORIC-101 to reverse taxane resistance was assessed in cell lines and xenograft models, and a phase 1 study (NCT03928314) was conducted in patients with advanced solid tumors to determine the dose, safety, and antitumor activity of ORIC-101 with nab-paclitaxel.

**Results:**

ORIC-101 reversed chemoprotection induced by glucocorticoids in vitro and achieved tumor regressions when combined with paclitaxel in both taxane-naïve and -resistant xenograft models. In the phase 1 study, 21 patients were treated in dose escalation and 62 patients were treated in dose expansion. All patients in dose expansion had previously progressed on a taxane-based regimen. In dose escalation, five objective responses were observed. A preplanned futility analysis in dose expansion showed a 3.2% (95% confidence interval, 0.4–11.2) objective response rate with a median progression-free survival of 2 months (95% confidence interval, 1.8–2.8) across all four cohorts, leading to study termination. Pharmacodynamic analysis of tissue and plasma showed GR pathway downregulation in most patients in cycle 1.

**Conclusions:**

ORIC-101 with nab-paclitaxel showed limited clinical activity in taxane-resistant solid tumors. Despite clear inhibition of GR pathway signaling, the insufficient clinical signal underscores the challenges of targeting a single resistance pathway when multiple mechanisms of resistance may be in play.

**Significance:**

Glucocorticoid receptor (GR) upregulation is a mechanism of resistance to taxane chemotherapy in preclinical cancer models. ORIC-101 is a small molecule GR inhibitor. In this phase 1 study, ORIC-101 plus nab-paclitaxel did not show meaningful clinical benefit in patients who previously progressed on taxanes despite successful GR pathway downregulation.

## Introduction

Glucocorticoids are steroid hormones secreted by the adrenal gland in response to hypothalamic stimuli in a circadian and stress-associated manner. They mediate their pleiotropic biological effects by binding to the intracellular glucocorticoid receptor (GR), which subsequently undergoes nuclear translocation and binds to glucocorticoid response elements in DNA to transcriptionally regulate various genes. Glucocorticoids regulate a myriad of physiologic functions, including metabolism, cell growth, apoptosis, differentiation, inflammation, mood, and cognitive function (1).

In cancer, GR is overexpressed in multiple epithelial cancers (2), including ovarian cancer, pancreatic ductal adenocarcinoma (PDAC), and triple-negative breast cancer (TNBC). Preclinical studies have implicated GR signaling in mediating resistance to both targeted therapies and conventional chemotherapies in these malignancies (3). In preclinical models of TNBC and ovarian cancer, genetic knockout of GR or pharmacologic inhibition of GR with first generation GR antagonist mifepristone enhances chemotherapy response (4–7).

ORIC-101 is a potent, selective, orally bioavailable small molecule GR antagonist with a more favorable cytochrome P450 inhibition profile than prior generation GR antagonists, making it particularly suitable for combination with taxane chemotherapy, which is primarily metabolized by cytochrome P450 2C8 (8). Therefore, we hypothesized that GR inhibition with ORIC-101 combined with taxane chemotherapy could increase the antitumor effect observed when compared to chemotherapy alone.

## Materials and Methods

### Preclinical studies

#### *In vitro* studies

For cell culture experiments, TNBC cell lines HCC1806 and Hs578T and PDAC cell lines BxPC3, SW1990, and PSN1 were maintained in RPMI-1640 medium, and ovarian cancer cell line COV362 in DMEM medium, supplemented with 10% fetal bovine serum (FBS), 1% penicillin/streptomycin and 2 mmol/L glutamine. TNBC cell line MDA-MB-231 was maintained in EMEM medium supplemented with 10% FBS, 1% Pen/Strep, 2 mmol/L glutamine, 1% non-essential amino acids (NEAA), and 1% sodium pyruvate. All cell lines were purchased from ATCC.

An ORIC-101 dose of 0.5 μmol/L was used in the *in vitro* studies. This dose achieved 90% modulation of GR target genes in peripheral blood mononuclear cells from healthy donors, corresponding to 90% of maximal efficacy of ORIC-101 in reversing chemotherapy resistance in preclinical models. A similar degree of GR pathway suppression was observed at ORIC-101 doses of 160 mg and higher in human healthy volunteers, supporting the *in vitro* ORIC-101 dose.

For colony formation assay experiments, cell lines were seeded at 10,000 or 20,000 cells/well in six-well plates with 2 mL of the above described culture media in duplicate. Twenty-four hours after plating, cells were treated with DMSO (Veh), 30 nmol/L dexamethasone (Dex), or 30 nmol/L dexamethasone plus 0.5 μmol/L ORIC-101 (Dex + 101) for 24 hours. DMSO (Veh), 100 nmol/L paclitaxel (PTX for TNBC cells), 100 nmol/L paclitaxel plus 100 nmol/L gemcitabine (Chemo for PDAC cells), or 100 nmol/L gemcitabine (Gem for ovarian cells) was added into the respective wells for 3 hours (ovarian line COV362) or 6 hours (PDAC and TNBC cell lines) and then media was removed to wash off chemo treatment. Fresh media with Veh, Dex, or Dex + 101 as indicated was added back to wells, media was replaced every 3 to 5 days and cells were cultured at 37°C incubator for 1 to 2 weeks until visible colonies formed. At the end of the assays, cells were stained with crystal violet and plates were scanned with the Celigo S Imaging Cytometer (Nexcelom, #200-BFFL-S) following manufacturer’s instructions to measure the percentage of confluency covered by the stained colonies in each well. Assay duration was 14 days for chemotherapy-treated cells and 8 days for untreated cells (Veh). Experiments were repeated three times and representative images are shown.

For apoptosis protection assays, 4,000 to 5,000 cells/well for each cell line were seeded in two sets of 96-well plates in 200 μL culture media as described above and incubated for 24 hours. Cells were treated with 100 nmol/L paclitaxel (TNBC cells), 100 nmol/L gemcitabine plus paclitaxel (PDAC cells), 100 nmol/L paclitaxel or gemcitabine (ovarian cells), vehicle, 30 nmol/L dexamethasone, 0.5 μmol/L of ORIC-101, or combinations thereof, as indicated in fresh culture media for 6 hours. Media was removed and fresh culture media with vehicle, 30 nmol/L dexamethasone, 0.5 μmol/L of ORIC-101, or combinations thereof was added into respective wells as indicated for an additional 42 hours (TNBC, PDAC cells) or 66 hours (ovarian cells). Then media was removed and 100 μL/well of PBS plus 100 μL/well of CellTiter-Glo Luminescent Cell Viability Assay reagent (Promega, #7573) or Caspase-Glo 3/7 Assay Systems reagent (Promega, #G8093) were added to plates 1 and 2, respectively. Plates were mixed on a shaker, incubated at room temperature for 12 minutes and read for luminescence on a Tecan Infinite F500 plate reader. To control for differences in cell number between treatments, data were normalized by dividing the Caspase-Glo 3/7 readings by the average CellTiter-Glo readings for each treatment. Fold-change in caspase activity was then calculated relative to vehicle control by dividing the normalized average of the Caspase-Glo 3/7 values by the value of the normalized average of the vehicle group. Data were plotted as mean ± SEM using GraphPad Prism 7.

#### *In vivo* studies

Female Envigo nude mice (Hsd:Athymic Nude-*Foxn1nu*) were used in all *in vivo* efficacy studies. HCC1806 cells (Source: ATCC) were grown in RPMI-1640 medium which was modified with 1% 100 mmol/L Na pyruvate, 1% 1 mol/L HEPES buffer, 1% of a 45% glucose solution and supplemented with 10% non-heat-inactivated FBS and 1% 100× penicillin/streptomycin/L-glutamine. Animals were implanted with HCC1806 cells in the mammary fat pad. Tumor size was measured by caliper twice weekly. Tumor volume (mm^3^) was estimated from caliper measurements as (*L* × *W*^2^)/2, where *L* and *W* are the respective orthogonal tumor length and width measurements (mm). Animals with tumors in excess of 2,000 mm^3^ were euthanized.

To evaluate ORIC-101 + paclitaxel efficacy in taxane-naïve HCC1806 TNBC xenografts, mice received either (i) paclitaxel intraperitoneally every 3 days for eight doses at a dose level of 20 mg/kg, (ii) 100 mg/L cortisol *ad libitum* in the drinking water, (iii) ORIC-101 75 mg/kg administered by oral gavage twice-daily, or (iv) combinations thereof. On days when both paclitaxel and ORIC-101 were administered, paclitaxel was administered approximately 5 hours after ORIC-101 or vehicle. The cortisol dose of 100 mg/L was chosen as it was found to be sufficient to simulate human cortisol levels and drive GR activation (8).

To evaluate ORIC-101 + paclitaxel efficacy in taxane-relapsed HCC1806 TNBC xenografts, mice began drinking 100 mg/L cortisol water 17 days prior to tumor cell inoculation and were provided cortisol continuously *ad libitum* in the drinking water for the entirety of the study. When the average tumor volume reached 200 mm^3^, all mice received paclitaxel intraperitoneally every 3 days for five doses at 15 mg/kg. After tumors regressed and then relapsed to approximately 200 mm^3^, mice were randomized into two groups. The first group was treated with paclitaxel intraperitoneally every 3 days for five doses. The second group received the same course of paclitaxel in combination with ORIC-101 at 150 mg/kg, administered by oral gavage once daily. On days when both drugs were administered, paclitaxel was administered approximately 5 hours after ORIC-101 or vehicle.

### Clinical trial design

This was a phase 1 open-label, dose escalation, and expansion study designed to evaluate the safety, tolerability, and antitumor activity of ORIC-101 in combination with nab-paclitaxel in patients with advanced solid tumors (ClinicalTrials.gov Identifier: NCT03928314).

During dose escalation, eligible patients were enrolled based on a standard 3+3 design (9) to evaluate ORIC-101 doses ranging from 80 to 240 mg once daily with food, administered on an intermittent or continuous daily dosing regimen in combination with 75 or 100 mg/m^2^ nab-paclitaxel as per standard of care.

The Recommended Phase 2 Dose (RP2D) was defined as the combination dose associated with the probability of dose-limiting toxicity (DLT) occurring in <30% of patients during the first treatment cycle (28 days). DLTs were graded according to the National Cancer Institute (NCI) Common Toxicity Criteria for Adverse Events (CTCAE) version 5.0 and defined in general as grade 3 or grade 4 adverse events (AE) considered related to study treatment. To be considered evaluable for DLT, patients must have received at least 75% of both ORIC-101 and nab-paclitaxel doses. Non-evaluable patients who discontinued treatment prior to completing cycle 1 for reasons other than toxicity (e.g., disease progression) could be replaced as per the 3+3 dose escalation rules. In addition, intrapatient dose escalation to a dose level previously deemed “safe” was allowed.

For the dose expansion, four cohorts of patients were enrolled based on tumor type: ovarian cancer, PDAC, TNBC, and a fourth cohort consisting of other solid tumors (OST) that met the eligibility criteria. Tumor assessments were performed every 8 weeks and included CT/MRI scans of the chest, abdomen, and pelvis (depending on tumor type), plus brain and/or bone scans (if applicable) using RECIST 1.1 (10). During dose expansion, images were also submitted for blinded independent central review (BICR).

An independent Data Monitoring Committee (DMC) monitored the conduct of the study to determine the overall safety and benefit-risk assessment of each expansion cohort, including prospectively planned interim analyses of futility to limit enrollment of participants to indications unlikely to benefit from the combination of ORIC-101 and nab-paclitaxel.

All patients or legal guardians provided written informed consent to participate and were treated until disease progression, unacceptable toxicity, or meeting other criteria for stopping treatment as per the study protocol. The study was conducted in accordance with the Declaration of Helsinki, Good Clinical Practice Guidelines, and relevant Institutional Review Board requirements.

### Patients

Eligible patients were ≥18 years old with advanced or metastatic solid tumors for whom no alternative effective standard therapy was available or for whom standard therapy was considered unsuitable or intolerable. During dose expansion, patients were required to have measurable disease at baseline as per BICR and have been previously treated with and progressed on a taxane-containing chemotherapy regimen. Other key inclusion criteria were an Eastern Cooperative Oncology Group (ECOG) performance status score of 0 or 1 with adequate bone marrow and organ function, a life expectancy of at least 3 months, and agreement and ability to undergo two on-study biopsies, one pretreatment and on-treatment during cycle 2. Key exclusion criteria included history of Cushing’s syndrome or adrenal insufficiency, requirement for chronic use of systemic corticosteroids, and in female patients, history of unexplained vaginal bleeding in the 8 weeks prior to the first dose of ORIC-101 or hormone replacement therapy.

### Study objectives

The primary objective in dose escalation was to identify the RP2D of ORIC-101 in combination with nab-paclitaxel based upon safety and tolerability, along with appropriate target therapeutic exposure. The primary objective in dose expansion was to evaluate the preliminary antitumor activity of ORIC-101 in combination with nab-paclitaxel based on objective response rate (ORR), defined as the best radiographic response as determined by RECIST 1.1 recorded from the start of study treatment until the end of treatment.

### Pharmacokinetic assessments

Blood samples were collected for analysis of plasma concentrations of ORIC-101 (and its metabolites) and paclitaxel, using validated bioanalytical methods. Pharmacokinetic parameters were determined using noncompartmental analysis (Phoenix WinNonLin software, Certara) and included maximum plasma concentration (C_max_) and area under the plasma concentration-time curve (AUC) from time zero up to the last quantifiable timepoint post-dose (AUC_0–t_).

### Pharmacodynamic assessments

Peripheral blood mononuclear cells (PBMC) were collected on multiple days and times throughout the first two cycles (1 cycle = 28 days), before and after dosing. PD modulation was assessed by RT-qPCR for housekeeping gene *RPL27* and GR target genes *FKBP5*, *GILZ* and *PER1*. These three GR target genes were selected for their consistent stimulation by GR and downregulation by ORIC-101 in preclinical studies of PDAC, TNBC and lung cancer cell lines and mouse models, and in the first-in-human single and multiple ascending dose studies of ORIC-101 administered to healthy subjects (11–13). Cortisol levels in blood were simultaneously measured at the time of PBMC collection.

In tumor biopsies obtained at screening, end of cycle 2, and end of treatment, GR protein status and GR pathway modulation were assessed as potential predictive biomarkers of ORIC-101. A proprietary IHC assay for GR protein status was developed and optimized for staining nuclear GR in the epithelial compartment of tumor tissue. GR protein levels were quantified by H-scores ranging from 0 to 300 and defined as (% tumor cells with weak staining) + 2× (% tumor cells with moderate staining) + 3× (% tumor cells with strong staining). Low GR expression was defined as *H*-score <100. Formalin-fixed, paraffin-embedded tumor blocks were sectioned with one slide H&E stained. Using the H&E slide as guiding slide, the tumor area on additional slides was then macrodissected, followed by RNA extraction and RNA sequencing at Q2 Solutions|EA Genomics. RNA sequencing data were aligned against the human Gencode 34 reference of the protein coding transcripts (14). A GR-specific activation signature, comprising of GR target genes *FKBP5*, *GILZ*, *PER1*, and *KLF9*, was quantitatively assessed in each tumor biopsy, relative to a distribution of expected GR activation built from RNA profiles of all archival and fresh tumor biopsies collected at any time during the ORIC-101 trial (11). Such a background distribution reflects the expected average *z*-scored expression of the four GR target genes within the reference population. GR activation in each tumor biopsy was then placed onto the reference distribution, by recentering and rescaling the expression of each target gene using the mean and standard deviation from the reference population, followed by averaging expression across the four target genes.

### Statistical assessments and sample size

During dose escalation, the sample size was based on a standard 3+3 design with three to six patients enrolled at each dose level. The number of dose escalations was dependent on the observed safety and available PK/PD data.

For the four expansion cohorts (ovarian, PDAC, TNBC, and OST), patients were enrolled under a multiple expansion cohort design, called the MUCE design (15), to determine whether ORIC-101 in combination with nab-paclitaxel had sufficient anticancer activity in each expansion cohort. Per the statistical analysis plan, patients would be enrolled in the four cohorts in parallel and planned interim analyses would be conducted based on prespecified reference and target response rates: the first interim analysis was to be conducted when half of the planned sample size for the fastest enrolling arm was reached and the second interim when the half value was reached for the slowest enrolling arm. If all cohorts enrolled patients at the same rate, there would be only one interim analysis. Through simulation studies, a maximum total of 132 patients were to be enrolled under MUCE if no futility stopping occurred at any interim analysis. For each expansion cohort, the maximum sample size and target response rate adjusted for the expected level of GR overexpression is shown in Supplementary Table S1. At the end of the trial, the Bayesian hierarchical model in the MUCE design was applied to the efficacy data to decide which cohorts would advance to further clinical development.

### Data availability

The data generated in this study are not publicly available due to concerns regarding patient privacy and consent but can be made available upon reasonable request to the corresponding author.

## Results

### Preclinical studies

The discovery of ORIC-101 was based upon structure-based modification of mifepristone and is detailed in previous work by Rew and colleagues (8). In brief, serial structure–activity relationship studies led to the discovery of a GR antagonist similar in potency to mifepristone (IC_50_ = 5.6 ± 2.6 nmol/L for ORIC-101; IC_50_ = 3.3 ± 0.6 nmol/L for mifepristone in cancer cells) but with significantly reduced androgen receptor (AR) agonism and improved cytochrome P450 (CYP) inhibition profiles.

**Figure 1:**
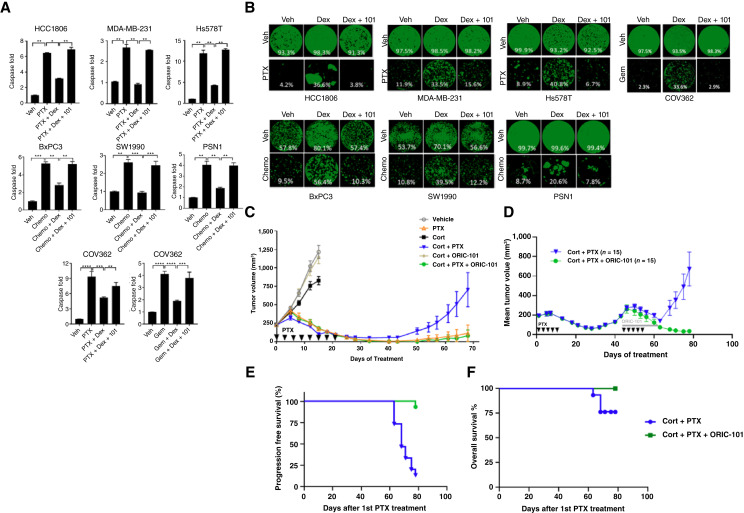
GR antagonism by ORIC-101 overcomes taxane resistance in preclinical models. **A** and **B,** Effect of ORIC-101 on dexamethasone-induced chemoprotection *in vitro* as measured by (**A**) caspase 3/7 apoptosis assays and (**B**) colony formation assays, in three TNBC lines (HCC1806, MDA-MB-231, Hs578T), three PDAC lines (BxPC3, SW1990, PSN1), and ovarian cancer line COV362. Veh: vehicle; Dex: 30 nmol/L dexamethasone; 101: 0.5 μmol/L ORIC-101; Chemo = 100 nmol/L paclitaxel + 100 nmol/L gemcitabine; PTX = 100 nmol/L paclitaxel; DTX = 100 nmol/L docetaxel; Gem = 100 nmol/L gemcitabine. **A,** The caspase activities of Veh were assigned as 1, and the values for other conditions were relative to Veh. One representative experiment with three biological repeats is shown (mean ± SEM). *, *P* < 0.05; **, *P* < 0.01; ***, *P* < 0.001 by one-way ANOVA using Tukey’s test to correct for multiple comparisons. **B,** One representative experiment with three biological repeats is shown. Numbers in white indicate the percentage of confluency quantitated by Celigo S. **C,** Effect of ORIC-101 on paclitaxel response in taxane-naïve HCC1806 TNBC xenografts in cortisol-treated mice. Mice were treated with paclitaxel 20 mg/kg by intraperitoneal injection every 3 days for a total of eight cycles (20 mg/kg, IP, Q3D × 8), cortisol (100 mg/L in drinking water, *ad libitum*) or ORIC-101 75 mg/kg by oral administration twice daily (75 mg/kg, PO, BID) starting on day 0 for the duration of the study. Arrowheads indicate PTX dosing days. Data are displayed as mean ± SEM. **D** and **E,** Effect of ORIC-101 in paclitaxel-relapsed HCC1806 TNBC xenografts in cortisol-treated mice. **D,** Animals were dosed with paclitaxel (15 mg/kg, IP, Q3Dx5) as indicated by the black arrowheads or ORIC-101 (150 mg/kg, PO, QD) as indicated by gray line. *n* = 15 in each group. Data is displayed as mean ± SEM. **E,** PFS, defined as the time elapsed between treatment initiation of the second cycle of paclitaxel and tumor progression or death from any cause, as a percentage of all animals in each cohort. A Kaplan–Meier plot was constructed to show the percentage of animals remaining in the study following treatment (*P* < 0.0001 by log-rank test). **F,** Overall survival, defined as the time elapsed between treatment initiation of the second cycle of paclitaxel and death from any cause as a percentage of all animals in each cohort, was not significantly different between the cohorts (by log-rank test).

Given the previously described association between GR activation and decreased sensitivity to taxanes, we first evaluated whether ORIC-101 could reverse resistance to various chemotherapeutic agents in preclinical cancer models. Across a variety of solid tumor cell lines (TNBC, ovarian cancer, and pancreatic cancer), exposure to the GR ligand dexamethasone induced chemoprotection to paclitaxel, docetaxel, and gemcitabine that was reversed by the addition of ORIC-101 (Fig. 1A and B). We then generated murine xenograft models implanted with taxane-naïve HCC1806 (TNBC) tumors to evaluate the effect of ORIC-101 on tumor growth *in vivo*. Tumors were grown in the absence or presence of cortisol, paclitaxel, and ORIC-101. As shown in Fig. 1C, cortisol administration significantly diminished the antitumor activity of paclitaxel in mice grown under conditions simulating human cortisol levels. The addition of ORIC-101 reversed cortisol’s effect on paclitaxel activity, resulting in sustained tumor growth inhibition relative to paclitaxel + cortisol. Finally, in a separate cohort of HCC1806-bearing mice, all tumors initially regressed but then relapsed following one cycle of paclitaxel treatment. When treated with paclitaxel alone, these taxane-relapsed tumors grew rapidly following the second cycle of paclitaxel treatment. Concurrent treatment with ORIC-101, however, significantly reduced tumor size and incidence (Fig. 1D) and improved PFS (Fig. 1E). Limited death events were observed in this study (Fig. 1F). These results supported the underlying hypothesis that GR antagonism could improve response to chemotherapy and warranted evaluation of ORIC-101 in a clinical setting.

### Patient disposition and demographics

A total of 83 patients were enrolled between May 2019 and September 2022. Twenty-one patients were enrolled during dose escalation and 62 in dose expansion at the RP2D across four cohorts: ovarian (*n* = 14), PDAC (*n* = 21), TNBC (*n* = 7), and other solid tumors (*n* = 20). The other solid tumors cohort encompassed patients with 10 different tumor types: anal, hormone receptor-positive breast, cervical, endometrial, esophageal, gastric, lung, prostate, salivary, and testicular cancer. Patient characteristics were consistent with an advanced refractory solid tumor patient population (Table 1), with a median of three prior lines of therapy. Patients in dose expansion were required to have progressed on prior taxane therapy or, in the case of ovarian cancer, relapsed within 6 months of platinum-taxane doublet therapy. Patients were not allowed systemic glucocorticoid administration for the duration of the study, although use of inhaled and topical steroids was allowed.

**Table 1:** Baseline characteristics and demographics

	Dose escalation	Dose expansion at the RP2D (160 mg ORIC-101 + 75 mg/m^2^ nab-paclitaxel)				Total (*N* = 83)
	80–240 mg ORIC-101 + 75–100 mg/m^2^ nab-pac (*n* = 21)	PDAC (*n* = 21)	OVARIAN (*n* = 14)	TNBC (*n* = 7)	OST (*n* = 20)	
Age, years						
Median (range)	56 (24–80)	63 (46–78)	69 (48–83)	60 (44–73)	61 (29–73)	70 (53–83)
Sex, *n* (%)						
Male	10 (47.6)	8 (38.1)	—	—	4 (20)	22 (26.5)
Female	11 (52.4)	13 (61.9)	14 (100)	7 (100)	16 (80)	61 (73.5)
Race, *n* (%)						
White	16 (76.2)	15 (71.4)	12 (85.7)	3 (42.9)	13 (65)	59 (71.1)
African American	1 (4.8)	2 (9.5)	0	3 (42.9)	0	6 (7.2)
Asian	3 (14.3)	3 (14.3)	2 (14.3)	0	3 (15)	11 (13.3)
Other	1 (4.8)	1 (4.8)	0	1 (14.3)	4 (20)	7 (8.4)
ECOG, *n* (%)						
0	7 (33.3)	2 (9.5)	7 (50)	0	3 (15)	19 (22.9)
1	14 (67.7)	19 (90.5)	7 (50)	7 (100)	17 (85)	64 (77.1)
Site of metastasis, *n* (%)						
Bone	4 (19.0)	1 (4.8)	2 (14.3)	1 (14.3)	5 (25)	13 (15.7)
Brain	1 (4.8)	0	0	0	0	1 (1.2)
Liver	10 (47.6)	12 (57.1)	8 (57.1)	2 (28.6)	12 (60	48 (57.8)
Lung	10 (47.6)	8 (38.1)	2 (14.3)	2 (28.6)	16 (80)	34 (41.0)
Lymph	9 (42.9)	6 (28.6)	9 (64.3)	5 (71.4)	7 (35)	36 (43.4)
Other	0	4 (19.0)	8 (57.1)	3 (42.9)	10 (50)	30 (36.1)
No. prior lines of therapy						
Median (range)	2.0 (1–8)	2 (1–5)	4 (1–6)	5 (3–12)	4 (1–8)	3 (1–12)
Prior anticancer surgery, *n* (%)	19 (90.5)	7 (33.3)	11 (78.6)	2 (28.6)	16 (80)	55 (66.3)
Prior radiation therapy, *n* (%)	10 (47.6)	3 (14.3)	2 (14.3)	2 (28.6)	9 (45)	26 (31.3)

Abbreviation: ECOG, Eastern Oncology Cooperative Group.

**Figure 2:**
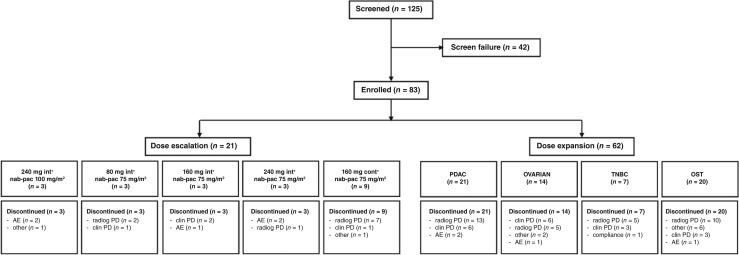
Study disposition flow diagram of patient disposition. Note: int, intermittent regimen of ORIC-101 administered 5 days on, 2 days off for 21 days out of 28-day cycles; cont, continuous regimen of ORIC-101 administered for 21 days out of 28-day cycles; AE, adverse event; clin, clinical; OST, other solid tumors; PD, progressive disease; radiog, radiographic.

Disease progression was the most common reason for treatment discontinuation in both dose escalation [66.6% (14/21) of patients] and dose expansion [79.0% (49/62) of patients]. Study patient disposition is presented in Fig. 2.

### Safety profile

The dose escalation portion of the study was designed to first evaluate intermittent administration (5 days on, 2 days off for 21 days) followed by continuous administration (21 days) of ORIC-101 in combination with 75 or 100 mg/m^2^ nab-paclitaxel on days 1, 8, and 15 of each 28-day cycle. Dose level 1 started with intermittent 240 mg ORIC-101 plus 100 mg/m^2^ nab-paclitaxel. However, at this dose level, one patient experienced DLTs of grade 4 neutropenia, grade 4 thrombocytopenia, and grade 5 hepatic failure. The grade 5 hepatic failure event was considered possibly related to drug, but also likely due to extensive liver metastases at baseline and rapid disease progression. A second patient had worsening grade 3 fatigue that also met criteria for DLT. Following discussion with the safety review committee, the study protocol was amended to reduce the starting doses of ORIC-101 to 80 mg and nab-paclitaxel to 75 mg/m^2^ and dose escalation was restarted. No subsequent DLTs were reported, and the combination RP2D was selected as 160 mg ORIC-101 on days 1 to 21 plus 75 mg/m^2^ nab-paclitaxel on days 1, 8, and 15 of each 28-day cycle with no growth factor support.

During dose escalation (*n* = 21), the most common treatment-emergent AEs reported in at least 15% of total patients were nausea (47.6%), fatigue (42.9%), diarrhea (38.1%), constipation (33.3%), AST increased (28.6%), anemia, decreased white blood cell count, vomiting (23.8% each), and abdominal pain, alopecia, ALT increased, blood alkaline phosphatase increased, decreased appetite, dyspnea, neutrophil count decreased (19% each; Supplementary Table S2). Serious AEs (SAE) were reported in 11 (52.4%) patients, with only two SAEs occurring in two patients each: small intestinal obstruction and pleural effusion. Five (23.8%) patients discontinued study treatment due to AEs.

During dose expansion at the RP2D (*n* = 62), 44 (71%) patients experienced AEs considered related to study treatment (TRAEs), with the most common being nausea (27.4%), fatigue (22.6%), anemia and vomiting (24.2% each), neutropenia (19.4%), diarrhea (12.9%), and alopecia (11.3%; Table 2). Treatment-related SAEs were reported in 2 (3.2%) patients (one case each of neutropenia and rash) and 4 (6.5%) discontinued study treatment due to AEs. One patient died of sepsis not related to study treatment.

**Table 2:** TRAEs occurring in at least 10% of dose expansion patients or grade 3 in severity treated at the RP2D

	PDAC (*n* = 21)		OVARIAN (*n* = 14)		TNBC (*n* = 7)		OST (*n* = 20)		Total (*N* = 62)	
Patients with at least one TRAE *n* (%)	All grades	Grade 3	All grades	Grade 3	All grades	Grade 3	All grades	Grade 3	All grades	Grade 3
Nausea	4 (19.0)	—	5 (35.7)	—	1 (14.3)	—	7 (35.0)	—	17 (27.4)	
Anemia	4 (19.0)	—	3 (21.4)	2 (14.3)	2 (28.6)	—	6 (30.0)	1 (5.0)	15 (24.2)	3 (4.8)
Vomiting	4 (19.0)	—	3 (21.4)	—	2 (28.6)	—	6 (30.0)	—	15 (24.2)	
Fatigue	2 (9.5)	—	6 (42.9)	2 (14.3)	1 (14.3)	1 (14.3)	5 (25.0)	—	14 (22.6)	3 (4.8)
Neutropenia	7 (33.3)	4 (19.0)	2 (14.3)	1 (7.1)	1 (14.3)	1 (14.3)	2 (10.0)	1 (5.0)	12 (19.4)	7 (11.3)
Diarrhea	2 (9.5)	—	4 (28.6)	—	—	—	2 (10.0)	—	8 (12.9)	—
Alopecia	—	—	4 (28.6)	—	—	—	3 (15.0)	—	7 (11.3)	
Leukopenia	2 (9.5)	1 (4.8)	1 (7.1)	1 (7.1)	1 (14.3)	1 (14.3)	1 (5.0)	—	5 (8.1)	3 (4.8)
Febrile neutropenia	1 (4.8)	1 (4.8)							1 (1.6)	1 (1.6)
AST increased	1 (4.8)	—	—	—	1 (14.3)	—	1 (5.0)	1 (5.0)	3 (4.8)	1 (1.6)
ALT increased	1 (4.8)	—	—	—	1 (14.3)	—	1 (5.0)	1 (5.0)	3 (4.8)	1 (1.6)
ALK PHOS increased	1 (4.8)	1 (4.8)	—	—	—	—	1 (5.0)	—	2 (3.2)	1 (1.6)
Hypokalemia	—	—	—	—	—	—	1 (5.0)	1 (5.0)	1 (1.6)	1 (1.6)
Hypophosphatemia	—	—	—	—	—	—	1 (5.0)	1 (5.0)	1 (1.6)	1 (1.6)
Rash	1 (4.8)	1 (4.8)	—	—	-	——	—	—	1 (1.6)	1 (1.6)
Peripheral neuropathy	1 (4.8)	1 (4.8)	—	—	1 (14.3)	—	—	—	1 (1.6)	1 (1.6)

Abbreviations: ALT, alanine aminotransferase; ALK PHOS, blood alkaline phosphatase; AST, aspartate aminotransferase; TRAE, treatment-related adverse events (related to ORIC-101 or nab-paclitaxel); AEs coded using MedDRA v22.0.

### Pharmacokinetic data

During dose escalation, pharmacokinetic data demonstrated ORIC-101 exposures increasing linearly with dose increase. At the RP2D of 160 mg ORIC-101 once daily for 21 days, a steady-state mean C_max_ (% CV) of 321 ng/mL (52.5) and AUC (% CV) of 966 h × ng/mL (42.6) were in line with the predicted human PK based on nonclinical studies and confirmed ORIC-101 achieved intratumoral exposures necessary for GR target coverage. There was no drug–drug interaction with nab-paclitaxel (16).

### Pharmacodynamic data

**Figure 3:**
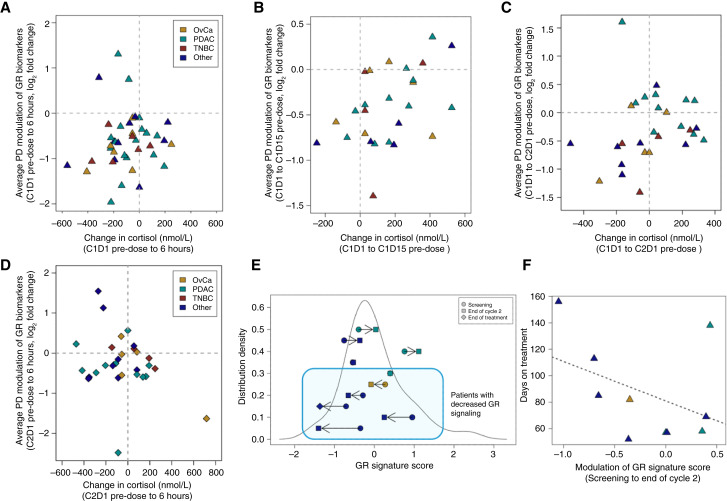
ORIC-101 pharmacodynamics in PBMCs and tumor specimens. **A,** Pharmacodynamic (PD) modulation (average expression of GR target genes *FKBP5*, *GILZ*, and *PER1*) on day 1 of cycle 1 as observed in 40 dose expansion patients with PBMC sample collection and cortisol assessment at C1D1 (pre-dose and 6 hours post-dose), and with pre-dose cortisol levels >200 nmol/L. Samples are colored by cohort. **B,** PD suppression from cycle 1 day 1 (pre-dose) to day 15 (pre-dose) as observed in 26 patients with PBMC sample collection and cortisol assessment at C1D1 pre-dose and C1D15 pre-dose, and with C1D1 pre-dose cortisol levels >200 nmol/L. **C,** PD suppression from cycle 1 day 1 (pre-dose) to cycle 2 day 1 (pre-dose) as observed in 28 patients with PBMC sample collection and cortisol assessment at C1D1 pre-dose and C2D1 pre-dose, and with C1D1 pre-dose cortisol levels >200 nmol/L. **D,** PD modulation on day 1 of cycle 2 as observed in 30 dose expansion patients with PBMC sample collection and cortisol assessment at C2D1 (pre-dose and 6 hours post-dose) and with pre-dose cortisol levels >200 nmol/L. **E,** On-treatment change in GR activation signaling in 10 patients (four dose escalation, six dose expansion) with paired tumor biopsies. The horizontal arrows indicate directionality of observed changes in GR activation as indicated by GR Signature Score (average expression of *FKBP5*, *GILZ*, *PER1*, and *KLF9*) from screening to end of cycle 2 or end of treatment, in the 10 patients. The solid line shows the expected distribution of GR activation from a reference cohort of archival and fresh tumor biopsies collected during this ORIC-101 trial. Patients with decreased GR signaling are highlighted in the boxed area. **F,** Association of on-treatment decrease in GR activation signaling with longer time on treatment.

Pharmacodynamic effects of ORIC-101 were assessed in PBMCs by RT-qPCR for GR target genes *FKBP5*, *GILZ*, and *PER1*, selected for their consistent stimulation by GR and reversal with ORIC-101 in preclinical and prior clinical studies (see “Materials and Methods”). At the RP2D, 92.5% (37/40) of patients with pre-treatment cortisol levels sufficient for GR stimulation (>200 nmol/L) showed a reduction in GR target gene expression in PBMCs 6 hours after initial ORIC-101 administration on day 1, suggesting potent and rapid target engagement (Fig. 3A). In pre-dose blood draws on day 15, GR target gene expression was already downregulated in 81% (21/26) of patients prior to that day’s ORIC-101 dose, indicating steady-state pathway suppression (Fig. 3B). This suppression was sustained to the beginning of cycle 2 in 64% (18/28) of patients (Fig. 3C) and confirmed by the lack of additional reduction in GR target gene expression in PBMCs 6 hours after ORIC-101 administration on day 1 of cycle 2 (Fig. 3D) contrary to suppression on day 1 of cycle 1 in 92.5% of patients (Fig. 3A). Paired tumor biopsies, collected during screening and towards the end of cycle 2 (1 cycle = 28 days), from 10 patients were characterized by RNA-sequencing. The same three GR target genes that had been measured in plasma as well as GR target gene *KLF9* were assessed in these tumors. There was a decrease in the average expression of these target genes in 6 out of the 10 patients (60%, Fig. 3E). Sustained GR inhibition as measured in the periphery and tumor was therefore achieved by ORIC-101 in the majority of patients throughout the first two cycles in the presence of rising cortisol levels (Fig. 3B and C). Among the 10 patients for which biopsies were available, there was a trend toward an inverse correlation between the magnitude of decline in GR activation signaling in the tumor during cycle 2 and the time the patient spent on study (Fig. 3F; Pearson correlation −0.42; *P* value 0.23), suggesting a possible association between the degree of clinical benefit from the therapeutic regimen with the degree of tumor GR pathway inhibition.

Eighty-seven pretreatment or archival biopsies were collected, with 83 (95%) having sufficient tumor cell content for GR protein quantification by immunohistochemistry. Moderate to high levels of nuclear GR protein (IHC *H*-score ≥100) were observed at baseline across all tumor types, especially ovarian cancer (in 100% of patients) and PDAC (96% of patients), making GR protein an unlikely biomarker of response. We did not see a clear, consistent change in GR expression as measured by IHC from screening to the end of cycle 2 among available biopsies.

### Antitumor activity

Among the 21 patients enrolled in dose escalation, 19 had a post-treatment radiographic tumor assessment and were considered evaluable. Twenty-six percent (5/19) of patients treated at varying doses of ORIC-101 in combination with 75 mg/m^2^ nab-paclitaxel had a partial response according to RECIST 1.1. Four responses were unconfirmed [endometrial cancer (80 mg intermittent), PDAC (160 mg continuous), lung (240 mg intermittent), and ER^+^ breast cancer (160 mg continuous)] and one response was confirmed [esophageal (240 mg intermittent)]. Three of these patients had previously progressed on a taxane-containing regimen (endometrial cancer, PDAC, and ER^+^ breast cancer; ref. 17). The patient with esophageal cancer who experienced a confirmed partial response had not previously been treated with a taxane-containing regimen, and thus the relative contribution of ORIC-101 to the clinical response was unclear There was no clear trend in on-treatment changes in intratumoral GR expression between responders and nonresponders.

In the dose expansion portion, the first interim analysis was triggered when the fastest enrolling arm (PDAC) reached 50% of the planned enrollment of 24 patients. At that point, a total of 35 patients comprised the efficacy analysis population, defined as patients with measurable disease at baseline and who had been followed for at least 12 weeks: ovarian (*n* = 7), PDAC (*n* = 12), TNBC (*n* = 4) and OST (*n* = 12) patients. Investigator-assessed ORR was 0% across all four cohorts (Table 3), which did not meet the target rate defined in the statistical analysis plan of ≥5% to warrant continuation of the study. Per DMC recommendation, enrollment into the PDAC cohort was stopped; however, since the ovarian, TNBC, and OST cohorts had not yet reached their respective halfway enrollment milestones, enrollment into those three cohorts was continued until the next fastest enrolling cohort (ovarian) reached its 50% enrollment goal.

**Table 3:** Antitumor activity at the RP2D

	PDAC (*N* = 21)	OVARIAN (*N* = 14)	TNBC (*N* = 7)	OST (*N* = 20)	TOTAL (*N* = 62)
Objective response rate (ORR): *n* (%; 95% CI)					
Interim analysis (*n* = 35)	0 (0.0–26.5)	0 (0.0–41.0)	0 (0.0–60.2)	0 (0.0–26.5)	0 (0.0–10.0)
Final analysis (*n* = 62)	0 (0.0–16.1)	1 (7.1)	0 (0.0–41.0)	1 (5.0)	2 (3.2)
				(0.1–24.9)	(0.4–11.2)
		(0.2–33.9)			
PFS: median (months; 95% CI)					
Interim analysis (*n* = 35)	1.7 (1.02–3.12)	2.02 (1.12–2.37)	NE (NE–NE)	1.87 (0.92–5.13)	1.81 (1.58–2.37)
Final analysis (*n* = 62)	2.1 (1.58–3.02)	2.4 (1.12–5.45)	6.3 (1.28–NE)	2.4 (0.99–3.71)	2.1 (1.77–2.76)

Abbreviation: NE, not evaluable.

At the time of the second analysis, a total of 62 patients were enrolled: ovarian (*n* = 14), PDAC (*n* = 21; nine additional patients had already been consented when the decision to terminate enrollment at the first interim analysis was made), TNBC (*n* = 7), and OST (*n* = 20). The final BICR-confirmed ORR was 7% [ovarian (*n* = 1)], 0% (PDAC), 0% (TNBC), and 5% [OST (*n* = 1)]. The patient with ovarian cancer who experienced an objective response was 65 years old who had received five lines of prior therapy in the metastatic setting. She had a partial response for 5.8 months and was progression-free for 8.5 months. The patient in the other solid tumor cohort with a partial response had esophageal cancer and was a 72-year-old male with four lines of prior therapy. He had a partial response for 3.6 months and was progression-free for 5.4 months. Median PFS (secondary endpoint) across the four cohorts was approximately 2 months, which was considered not clinically meaningful (Table 3), and the study was terminated.

## Discussion

In this phase 1b study of ORIC-101 in patients with advanced solid tumors, we tested the hypothesis that taxane-resistant cancers could potentially be resensitized to nab-paclitaxel by concurrent administration of a highly potent GR antagonist, ORIC-101. The ORIC-101 and nab-paclitaxel combination was demonstrated to be safe and well-tolerated. Despite the promising activity of the combination in cell lines and xenograft models, a preplanned futility analysis for the dose expansion portion of the clinical study indicated that the response rate did not meet criteria for continuation, and the study was halted.

In the initial phase 1 study of another GR antagonist, relacorilant (Corcept Therapeutics), in combination with nab-paclitaxel, 3/57 (5%) response-evaluable patients with various tumor types achieved an objective response after progressing on prior taxane for metastatic disease (18). Subsequently, a single arm, open label, phase 3 study of relacorilant in combination with nab-paclitaxel in patients with metastatic pancreatic cancer was discontinued due to lack of meaningful clinical benefit (6% response rate) shown at the planned interim analysis based on 31 patients. Separately, a randomized phase 2 trial of relacorilant plus nab-paclitaxel versus nab-paclitaxel monotherapy was performed in an earlier line patient population with platinum-resistant/refractory ovarian cancer; that study demonstrated a modest, non-statistically significant improvement in progression-free survival (PFS) when relacorilant was given intermittently in combination with nab-paclitaxel versus nab-paclitaxel alone (5.6 vs. 3.8 months; ref. 19). However, the ORRs between the intermittent combination arm and the nab-paclitaxel monotherapy arm were similar (35.7% vs. 35.8%, respectively). Intermittently dosed relacorilant is currently being tested in combination with nab-paclitaxel in a randomized phase 3 trial in platinum-resistant ovarian cancer.

In contrast to the relacorilant trials, our study mandated prior progression on a taxane as an inclusion criterion in dose expansion It is possible that GR signaling may have a more central biological role in chemotherapy resistance in taxane-naïve relative to taxane-resistant patients. A multitude of diverse acquired resistance mechanisms have been identified against taxane chemotherapy, including those unrelated to GR signaling (20). For example, alterations in tubulin or its associated signaling pathways have been implicated in resistance to taxanes, which induce cell cycle arrest via microtubule stabilization (21). One study examining tumor specimens from 41 patients with advanced ovarian cancer reported significantly altered expression of the tubulin isoforms in patients with taxane-resistant disease relative to taxane-sensitive disease (22). In addition, some taxane-resistant epithelial ovarian cancers express higher levels of the MAPK pathway protein SYK, which is hypothesized to counteract the stabilization of microtubules by taxanes (23).

The relative importance of GR signaling in primary versus acquired resistance has been observed in other diseases. In prostate cancer, GR signaling has been implicated as a mechanism of resistance to androgen receptor pathway inhibitors (ARPI), such as enzalutamide and apalutamide (24, 25). In patients with metastatic castration-resistant prostate cancer (mCRPC), ORIC-101 was administered in combination with enzalutamide in an attempt to overcome this therapeutic resistance and restore ARPI sensitivity in a phase 1b study. In that trial, consisting mostly of patients with mCRPC following frontline treatment with enzalutamide (i.e., relatively early in their treatment course), tumors already displayed significant heterogeneity with a plethora of resistance mechanisms to ARPI which included but was not limited to GR upregulation. This indicates therapeutic benefit from a GR antagonist such as ORIC-101 may be restricted to an earlier disease setting when resistance mechanisms are fewer and more homogeneous (26).

One key strength of our trial is the comprehensive biomarker data analysis based on serial tumor and blood samples that allowed longitudinal assessment of GR pathway activity and cortisol levels and may help inform development of other anti-GR strategies. Limitations of our study are its relatively small sample size that, combined with the heterogeneous tumor types involved in the study, may confound assessments of histology-specific effects of GR antagonism. It is possible that a single-histology trial in patients who have not yet progressed on taxanes, like the ongoing randomized phase 3 trial of relacorilant and nab-paclitaxel in ovarian cancer, is needed to capture signals of clinical efficacy; we eagerly await the results. In addition, it is possible that variation in patient-specific dexamethasone metabolism or germline genetic variations in the GR receptor can influence the relative benefit of ORIC-101. Ultimately, despite compelling preclinical data and clinical demonstration of GR pathway signaling inhibition, GR antagonism as an anticancer strategy may have only modest clinical benefit in overcoming taxane resistance.

In conclusion, while safe and tolerable, the overall benefit-risk profile of the ORIC-101 and nab-paclitaxel combination did not support further development of ORIC-101 for the treatment of advanced, heavily pretreated solid tumors.

## Supplementary Material

Supplementary Table 1 Supplementary Table 1. Statistical Assumption and Sample Size Estimates for MUCE Design

Supplementary Table 2 Supplementary Table 2. TEAEs Occurring in at Least 15% of Dose Escalation Patients

## References

[1] Azher S , Azami O, Amato C, McCullough M, Celentano A, Cirillo N. The non-conventional effects of glucocorticoids in cancer. J Cell Physiol 2016;231:2368–73. 27115293 10.1002/jcp.25408

[2] Block TS , Murphy TI, Munster PN, Nguyen DP, Lynch FJ. Glucocorticoid receptor expression in 20 solid tumor types using immunohistochemistry assay. Cancer Manag Res 2017;9:65–72. 28293120 10.2147/CMAR.S124475 PMC5345989

[3] Volden PA , Conzen SD. The influence of glucocorticoid signaling on tumor progression. Brain Behav Immun 2013;30:S26–31. 23164950 10.1016/j.bbi.2012.10.022 PMC3987853

[4] Hou WJ , Guan JH, Dong Q, Han YH, Zhang R. Dexamethasone inhibits the effect of paclitaxel on human ovarian carcinoma xenografts in nude mice. Eur Rev Med Pharmacol Sci 2013;17:2902–8. 24254559

[5] Kroon J , Puhr M, Buijs JT, van der Horst G, Hemmer DM, Marijt KA, . Glucocorticoid receptor antagonism reverts docetaxel resistance in human prostate cancer. Endocr Relat Cancer 2016;23:35–45. 26483423 10.1530/ERC-15-0343 PMC4657186

[6] Skor MN , Wonder EL, Kocherginsky M, Goyal A, Hall BA, Cai Y, . Glucocorticoid receptor antagonism as a novel therapy for triple-negative breast cancer. Clin Cancer Res 2013;19:6163–72. 24016618 10.1158/1078-0432.CCR-12-3826 PMC3860283

[7] Stringer-Reasor EM , Baker GM, Skor MN, Kocherginsky M, Lengyel E, Fleming GF, . Glucocorticoid receptor activation inhibits chemotherapy-induced cell death in high-grade serous ovarian carcinoma. Gynecol Oncol 2015;138:656–62. 26115975 10.1016/j.ygyno.2015.06.033 PMC4556542

[8] Rew Y , Du X, Eksterowicz J, Zhou H, Jahchan N, Zhu L, . Discovery of a potent and selective steroidal glucocorticoid receptor antagonist (ORIC-101). J Med Chem 2018;61:7767–84. 30091920 10.1021/acs.jmedchem.8b00743

[9] Storer BE . Design and analysis of phase I clinical trials. Biometrics 1989;45:925–37. 2790129

[10] Eisenhauer E , Therasse P, Bogaerts J, Schwartz LH, Sargent D, Ford R, . New response evaluation criteria in solid tumours: revised RECIST guideline (version 1.1). Eur J Cancer 2009;45:228–47. 19097774 10.1016/j.ejca.2008.10.026

[11] Pankov A , Zhou H, Barkund S, Hegde G, Narayanan P, Kabbarah O, . Abstract 4120: ORIC-101 comprehensively inhibits glucocorticoid pathways to overcome therapeutic resistance in pan-cancer models. Cancer Res 2020;80(Suppl 16):4120.

[12] Zhou H , Ye Q, Pankov A, Kong W, Barkund S, Friedman LS, . Abstract P6-03-24: ORIC-101 robustly inhibits the glucocorticoid pathway and overcomes chemoresistance in triple-negative breast cancer. Cancer Res 2020;80(Suppl 4):P6-03–24.

[13] Daemen A , Pankov A, Barkund S, Zhou H, Duff M, Johnson A, . Biomarker results supporting selection of RP2D from a phase 1b study of ORIC-101, a glucocorticoid receptor antagonist, in combination with nab-paclitaxel in patients with advanced solid tumors. J Clin Oncol 2021;39(Suppl 15):3110.

[14] Frankish A , Diekhans M, Ferreira A-M, Johnson R, Jungreis I, Loveland J, . GENCODE reference annotation for the human and mouse genomes. Nucleic Acids Res 2019;47:D766–73. 30357393 10.1093/nar/gky955 PMC6323946

[15] Lyu J , Zhou T, Yuan S, Guo W, Ji Y. MUCE: Bayesian hierarchical modelling for the design and analysis of phase 1b multiple expansion cohort trials. J R Stat Soc Ser C Appl Stat 2023;72:649–69.

[16] Xu R , Wu YS, Duff M, Johnson AD, Multani PS, Chow Maneval E. Abstract 1053416: Pharmacokinetic evaluations from a phase 1b investigational study of ORIC-101, a glucocorticoid receptor antagonist, in combination with nab-paclitaxel in patients with advanced solid tumors. Philadelphia (PA): Proc of 2021 AAPC PharmSci 360; 2021.

[17] Abdul-Karim RM , Tolcher AW, Kummar S, Patel MR, Spira AI, Jauhari S, . Initial results from a phase 1b study of ORIC-101, a glucocorticoid receptor antagonist, in combination with nab-paclitaxel in patients with advanced solid tumors. J Clin Oncol 2021;39(Suppl 15):2553.

[18] Munster PN , Greenstein AE, Fleming GF, Borazanci E, Sharma MR, Custodio JM, . Overcoming taxane resistance: preclinical and phase 1 studies of relacorilant, a selective glucocorticoid receptor modulator, with nab-paclitaxel in solid tumors. Clin Cancer Res 2022;28:3214–24. 35583817 10.1158/1078-0432.CCR-21-4363 PMC9662918

[19] Colombo N , Van Gorp T, Matulonis UA, Oaknin A, Grisham RN, Fleming GF, . Relacorilant + nab-paclitaxel in patients with recurrent, platinum-resistant ovarian cancer: a three-arm, randomized, controlled, open-label phase II study. J Clin Oncol 2023;41:4779–89. 37364223 10.1200/JCO.22.02624 PMC10602497

[20] Maloney SM , Hoover CA, Morejon-Lasso LV, Prosperi JR. Mechanisms of taxane resistance. Cancers (Basel) 2020;12:3323. 33182737 10.3390/cancers12113323 PMC7697134

[21] Orr GA , Verdier-Pinard P, McDaid H, Horwitz SB. Mechanisms of taxol resistance related to microtubules. Oncogene 2003;22:7280–95. 14576838 10.1038/sj.onc.1206934 PMC4039039

[22] Mozzetti S , Ferlini C, Concolino P, Filippetti F, Raspaglio G, Prislei S, . Class III beta-tubulin overexpression is a prominent mechanism of paclitaxel resistance in ovarian cancer patients. Clin Cancer Res 2005;11:298–305. 15671559

[23] Yu Y , Gaillard S, Phillip JM, Huang TC, Pinto SM, Tessarollo NG, . Inhibition of spleen tyrosine kinase potentiates paclitaxel-induced cytotoxicity in ovarian cancer cells by stabilizing microtubules. Cancer Cell 2015;28:82–96. 26096845 10.1016/j.ccell.2015.05.009 PMC5257279

[24] Isikbay M , Otto K, Kregel S, Kach J, Cai Y, Vander Griend DJ, . Glucocorticoid receptor activity contributes to resistance to androgen-targeted therapy in prostate cancer. Horm Cancer 2014;5:72–89. 24615402 10.1007/s12672-014-0173-2 PMC4440041

[25] Puhr M , Hoefer J, Eigentler A, Ploner C, Handle F, Schaefer G, . The glucocorticoid receptor is a key player for prostate cancer cell survival and a target for improved antiandrogen therapy. Clin Cancer Res 2018;24:927–38. 29158269 10.1158/1078-0432.CCR-17-0989

[26] Abida W , Hahn AW, Shore N, Agarwal N, Sieber P, Smith MR, . Phase I study of ORIC-101, a glucocorticoid receptor antagonist, in combination with enzalutamide in patients with metastatic castration-resistant prostate cancer progressing on enzalutamide. Clin Cancer Res 2024;30:1111–20. 38226958 10.1158/1078-0432.CCR-23-3508 PMC10947849

