# Supplementary figures and images for: ORIC-101, a Glucocorticoid Receptor Antagonist, in Combination with Nab-Paclitaxel in Patients with Advanced Solid Tumors
Source: Cancer Res Commun. 2024 Sep 13;4(9):2415–26. doi: 10.1158/2767-9764.CRC-24-0115 (PMC11396014; doi:10.1158/2767-9764.CRC-24-0115)

## Slide 1
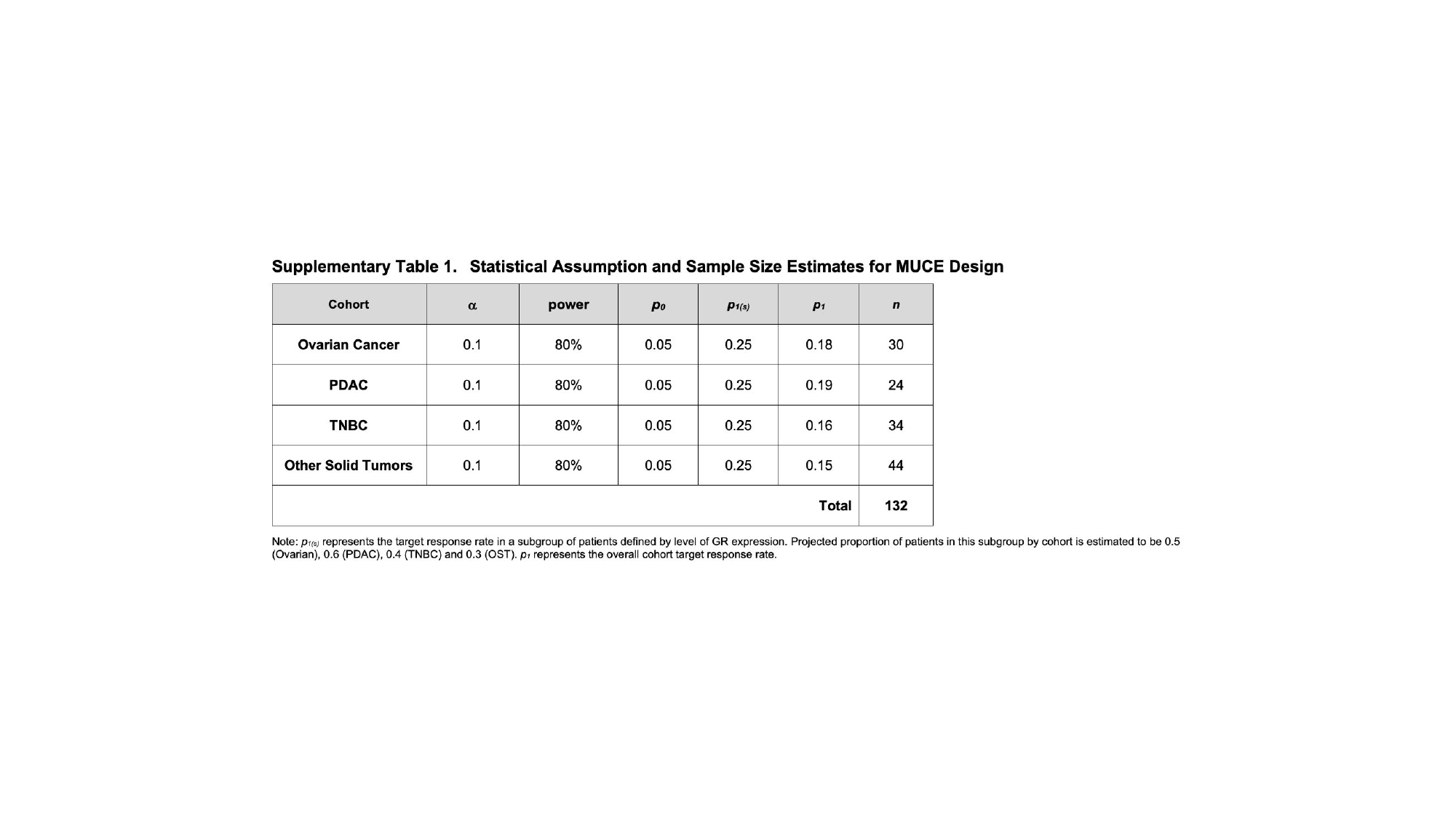

Supplement: Supplementary Table 1 — Statistical Assumption and Sample Size Estimates for MUCE Design [file crc-24-0115_supplementary_table_1_suppst1.pptx]

## Slide 1
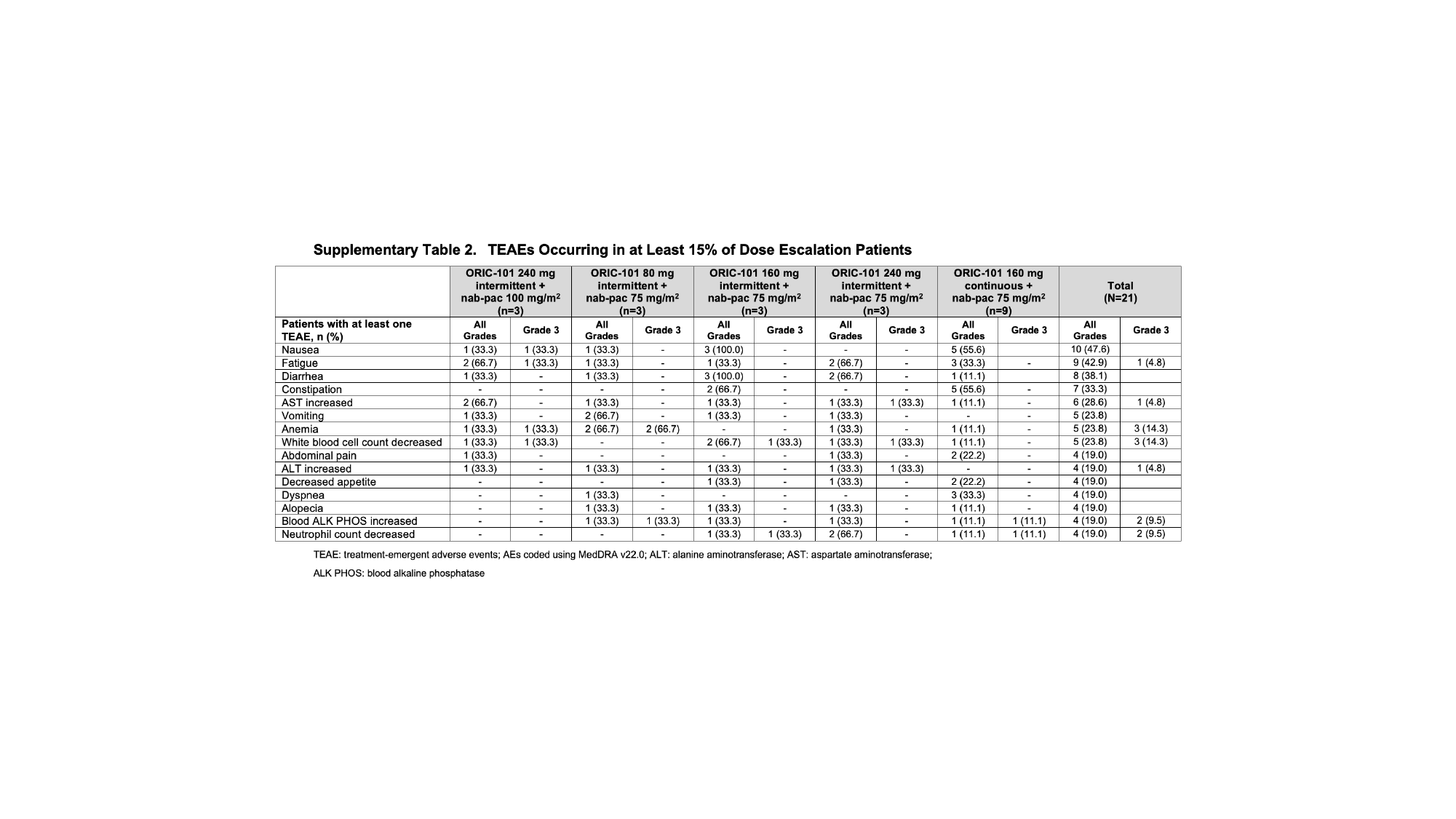

Supplement: Supplementary Table 2 — TEAEs Occurring in at Least 15% of Dose Escalation Patients [file crc-24-0115_supplementary_table_2_suppst2.pptx]
